# The effects of continuous nursing via the WeChat platform on neonates after enterostomy: a single-centre retrospective cohort study

**DOI:** 10.1186/s12912-023-01177-0

**Published:** 2023-01-12

**Authors:** Lijuan Wu, Ying Lin, Ruiyun Xue, Bin Guo, Jianxi Bai

**Affiliations:** Department of Pediatric Surgery, Fujian Children’s Hospital (Fujian Branch of Shanghai Children’s Medical Center), College of Clinical Medicine for Obstetrics & Gynecology and Pediatrics, Fujian Medical University, Fujian Maternity and Child Health Hospital, Fuzhou, 350001 P. R. China

**Keywords:** Continuous nursing, WeChat platform, Neonatal enterostomy

## Abstract

**Background:**

Temporary enterostomy is an effective treatment for various neonatal intestinal diseases. However, family caregivers find it challenging to provide the required nursing care. Nursing management is very important for reducing parents’ anxiety and improving the patients’ quality of life. This research aimed to compare the effects of continuous nursing using the WeChat platform with traditional nursing for neonates after enterostomy.

**Methods:**

Neonates who underwent enterostomy from January 2014 to December 2020 in our hospital were retrospectively analysed. The patients were divided into the traditional nursing group and the continuous nursing group. The peri-stomal skin was evaluated with the DET scale. The mental status of the families was evaluated with the SAS and SDS.

**Results:**

There were 143 patients in the traditional nursing group (TG) and 165 in the continuous nursing group (CG). The mean weight was 2.7 ± 0.6 kg in TG and 2.8 ± 0.5 kg in CG. The mean age at surgery was 4.9 ± 7.3 d in TG and 4.8 ± 7.55 d in the CG. No statistically significant differences between the two groups were found in the demographic information. The continuous nursing group had an obviously lower DET score for the peri-stomal skin than the traditional nursing group (*P* = 0.003). Three months after discharge from the hospital, the continuous nursing group replaced 7.2 ± 1.8 ostomy bags every week, significantly less than the traditional nursing group (*P* = 0.002). Three months after discharge, the continuous nursing group had better SAS and SDS scores than the traditional nursing group.

**Conclusions:**

Continuous nursing based on WeChat can effectively improve the quality of life of neonates after enterostomy. Family members can also receive proper psychological counselling to relieve their anxiety and depression.

## Background

Neonatal enterostomy refers to the surgical formation of an opening through the abdominal wall into the intestine as a temporary artificial anus and is used to rescue various neonatal intestinal diseases, such as necrotizing enterocolitis, anorectal malformation and intestinal atresia [1, 2]. A neonatal stoma is usually a temporary stoma, and the patients will undergo closure 3-6 months later. In general, the patients are discharged after their intestinal functions recover and their food intake reaches physiological requirements. In the following period, home is the main venue of nursing care and parents are the major caregivers [3].

Due to a lack of related experience, caregivers often find it difficult to learn ostomy care while in the hospital. Inappropriate ostomy care may lead to peristomal skin complications, which undermines the quality of life of the patients and increases the anxiety of their families [4, 5]. Providing a reasonable and efficient model of continuous nursing management after neonatal enterostomy is a significant issue that urgently needs to be addressed clinically. WeChat, the most popular instant messaging platform in China, has a low cost and high speed, supports face-to-face communication and has great value in providing continuous nursing services [6–8]. A single-centre retrospective cohort study was performed to analyse the effects of continuous nursing based on the WeChat platform for neonates after enterostomy.

## Methods

### Subjects

Neonates who underwent enterostomy from January 2014 to December 2020 in our hospital were retrospectively analysed. Our hospital started accumulating experience in continuous nursing based on the WeChat platform in January 2017. Patients admitted from January 2017 to December 2020 (the continuous nursing group) and from January 2014 to December 2016 (the traditional nursing group) were compared. All of the patients’ families completed the Self-Rating Anxiety Scale (SAS) on the first day after the operation and during re-examination 3 months after discharge. This study was approved by the ethics committee of our hospital (No. 2020YJ177) and strictly adhered to the tenets of the Declaration of Helsinki [9].

### Inclusion and exclusion criteria

The inclusion criteria were patients who undergo enterostomy during the neonatal period. The exclusion criteria were patients complicated with serious congenital heart disease or severe hepatic or renal insufficiency; patients who died after the operation; the parents were incapable of reading independently; no communication device was available or information communication was blocked; or families were unwilling to receive continuous nursing services or participate in the study.

### Nursing methods

#### Traditional nursing

During the inpatient stay, the nurses offered regular medical service to the patients, and the attending doctors explained to their families the disease-related knowledge, the surgical procedures, and matters requiring their attention after the operation. Nursing personnel offered the families regular nursing care guidance, including diet management, measurement of the weight and volume of stool, management of the peristomal skin, replacement of the ostomy bag and situations requiring emergency treatment. A patient file and a follow-up record were created for each patient. The patient was followed-up 1 week, 1 month, 2 months and 3 months after discharge.

#### Continuous nursing

After the operation, the nurses-in-charge instructed the patients’ families on how to useWeChat correctly and skilfully and join the WeChat continuous nursing group (named “Ostomy Angel”), in which continuous nursing was provided after discharge. The continuous nursing WeChat group consisted of five main members, including one attending doctor and four nurses with at least 5 years of working experience. Among them, two nurses were responsible for instructing the families in ostomy care during the inpatient stay until they were fully capable. The other two nurses were responsible for collecting information, including the patient’s name, sex, age, contact, time of enterostomy, location and type of stoma, family details, and postoperative complications. The attending doctor acted as an adviser.

The main tasks of the continuous nursing WeChat group had 3 parts: health education, questions and answers and teamwork. First, the group regularly sent messages on health education such as nursing care for the stoma, feeding of the infants, ways to handle common issues and provide first aid, postoperative psychological guidance, and other aspects related to rehabilitation. The families were able to learn independently at home.

Second, one medical personnel was on duty daily, staying online in the WeChat group during 12:00–15:00 and 18:00–21:00 to answer questions from the patients’ families. In addition, the patients’ parents were required to upload videos of the process of replacing the bag and pictures of the peristomal skin weekly, so that the medical personnel could closely follow the status of the patient’s stoma and correct any nursing problem the families’ encountered. Any patients’ families who failed to update their status for 2 weeks were separately followed via face-to-face communications, during which the medical personnel inquired about stoma nursing and peristomal skin conditions and offered guidance.

Third, to create a team atmosphere, the group members were encouraged to actively communicate with each other via the WeChat group in daily life, share their experience in nursing care and discuss their physical pain and mental pressure. Meanwhile, the medical personnel used guidance, explanations, encouragement, and other supportive methods to help the families and enliven the team atmosphere.

### Measures

#### Peristomal skin DET scale

Three symptoms, namely, discoloration (D), erosion/ulceration (E) and tissue overgrowth (T), are assessed in terms of the size of the affected peristomal skin area and its severity [10, 11]. The score for each symptom equals the score for the size of the affected area plus the score for the damage severity and ranges from 0 to 5 points. The score for the size of the affected area ranges from 0 to 3 points and score of damage severity ranges from 0 to 2 points. The DET total score equals the D score plus the E score plus the T score, ranging from 0 to 15 points. The score is categorized into four levels of severity: none (DET = 0 points), mild (DET = 1–3 points), moderate (DET = 4–6 points) and severe (DET = 7–15 points).

#### Self-rating Anxiety Scale (SAS)

Zung’s self-rating anxiety scale (SAS) [12] was adopted, which is widely applied clinically because of its high reliability and validity. Fifteen items are described as negators, and they are rated 1 to 4 based on the occurrence frequency of the symptoms. Five items are described as affirmatives, and the reverse rating method (4 to 1) is used to give a score based on the occurrence frequency of the symptoms. The scores of all of the items are added to obtain the total score. The total score is multiplied by 1.25 and then rounded off to obtain the standard score, and the average value of the standard score is 50. A score < 50 means normal, 50–59 mild anxiety, 60–69 moderate anxiety and ≥ 70 severe anxiety.

#### Self-rating Depression Scale (SDS)

Zung’s self-rating depression scale (SDS) [13] was adopted, which is widely applied clinically because of its high reliability and validity. The scale consists of 20 items, including 10 negative symptoms and 10 positive symptoms, with each question representing characteristics of depression. All of the items together can reflect feelings, symptoms of physical discomfort, mental activities and behavioural and psychological symptoms of depression, and the score is divided into four categories. The rating method 1 to 4 is used according to the occurrence frequency of positive symptoms, while the reverse rating method (4 to 1) is used to give a score based on the occurrence frequency of negative symptoms. The score is multiplied by 1.25 and then rounded off to obtain the standard score, with 41 being the ceiling score and 53 being the standard score. A higher score indicates a greater tendency towards depression.

### Statistical analysis

The statistical software SPSS 17.0 was used for analysis. Quantitative data are expressed as the mean value ± standard deviation and were subjected to an independent-sample t test for statistical analysis; qualitative data were compared between groups using the chi-square test. *P* < 0.05 indicated that the difference was statistically significant.

## Results

A total of 165 patients were enrolled in the continuous nursing group, and 143 were enrolled in the traditional nursing group. The demographic characteristics of patients in the two groups had no statistically significant differences (Table 1). During the 3-month follow-up of all of the patients after discharge, the traditional nursing group replaced 18.5 ± 3.5 ostomy bags every week, while the continuous nursing group replaced 7.2 ± 1.8 ostomy bags every week (*P* = 0.002).

**Table 1:** Demographic characteristics of patients in the two groups

Item	Continuous nursing group	Traditional nursing group	*P* value
Number of cases	165	143	
Age of the mother	26.9 ± 3.8	26.4 ± 3.6	0.23
Parity of the mother			
1	112	96	0.88
2	42	39	
3+	11	8	
Male/Female	101/64	96/47	0.280
Weight (kg)	2.7 ± 0.6	2.8 ± 0.5	0.384
Age (day)	4.9 ± 7.3	4.8 ± 7.5	0.712
Location of stoma			
Ileum	122	105	0.919
Transverse colon	43	38	
Location of family			
Countryside	108	95	0.857
Urban area	57	48	
Educational background of the parents			
Undergraduate and higher	65	57	0.458
High school-undergraduate	85	67	
High school and lower	15	19	

The continuous nursing group had a lower score than the traditional nursing group on the peristomal skin DET scale (*P* < 0.05). The peristomal skin of patients in the continuous nursing group was mostly healthy, while that in the traditional nursing group was mostly moderately or severely affected, and the difference was statistically significant (Table 2).

**Table 2:** Peristomal skin DET score three months after discharge

Item	Continuous nursing group	Traditional nursing group	*P* value
DET score	2.3 ± 1.4	6.8 ± 4.5	0.003
Number of patients in each score range			
0 point	132	33	0.000
1–3 points	20	35	
4–6 points	13	40	
7–15 points	0	35	

The two groups had no significant difference in SAS score or SDS score on the first day after the operation. Compared with the first day after the operation, the continuous nursing group had a much lower SAS score and SDS score 3 months after discharge, and the difference was statistically significant. In contrast, the SAS and SDS scores in the traditional nursing group increased, but the differences in thier scores were not statistically significant. For the SAS score and SDS score 3 months after discharge, the continuous nursing group considerably outperformed the traditional nursing group and the difference was statistically significant (*P* < 0.05) (Table 3).

**Table 3:** Mental status of the families of patients in the two groups on the first day after the operation and three months after discharge

Item	Continuous nursing group	Traditional nursing group	*P* value
SAS score			
1st day after the operation	63.8 ± 12.5	60.3 ± 13.5	0.813
3 months after discharge	48.1 ± 11.3^*^	65.8 ± 14.7	0.017
SDS score			
1st day after the operation	55.6 ± 12.3	54.3 ± 11.4	0.876
3 months after discharge	40.1 ± 9.6^*^	56.5 ± 14.6	0.012

^*^P < 0.05 compared to the score on the first day after the operation

## Discussion

Nursing care for neonatal stoma is critical for reducing the incidence of peristomal skin complications [14–16]. However, currently in China, medical services are concentrated in hospitals. As patients leave the hospital, the medical service relationship between hospitals and patients ends accordingly [17]. The majority of Chinese households are in the countryside, where access to medical care is limited and most rural medical centres are unable to provide professional stoma nursing services for neonates [18, 19]. In addition, most families that have limited educational backgrounds cannot fully master the knowledge and skills for stoma nursing during the short inpatient stay and only have access to their local township or community hospital for consultation when encountering nursing problems after discharge, where they can only receive nonprofessional nursing support. Consequently, patients tend to visit the hospital only in cases of severe complications, which seriously undermines the neonate’s health and affects the quality of closure of the ostomy. As found in this study, 3 months after discharge, most patients in the traditional nursing group had unhealthy peristomal skin, and only 33 patients (23.1%) were healthy. Therefore, continuous follow-up and nursing guidance after discharge for neonatal stoma is critical.

Continuous nursing extends the high-level and high-quality nursing service and psychological support in hospitals to patients’ families to ensure that high-quality treatment and nursing proceed at home without being interrupted, which can effectively address the issue of insufficient support for patients’ families after discharge and improve the quality of home nursing [20, 21]. Previously, continuous nursing mainly took the form of telephone follow-up, outpatient follow-up and family visits, but each of these has limitations [22]. Telephone follow-up, athough it is easy to conduct, is limited to verbal communications, which cannot offer guidance through direct visual images and cannot specifically identify the perceptions of the families, thus making it difficult to transmit information accurately. Outpatient follow-up and family visits, athough supporting direct face-to-face guidance, are difficult to conduct due to time costs, economic costs and labor costs. Therefore, exploring a more reasonable and efficient mode of continuous nursing management after neonatal enterostomy is an important issue clinically.

WeChat, as the most popular instant messaging platform in China, has a low cost, is convenient, and supports for face-to-face communication. Health education based on the WeChat platform offers a new approach to continuous nursing [23] and it has the following advantages. First, with continuous nursing based on the WeChat platform, medical personnel can answer questions in a timely manner, which improves the timeliness and effectiveness of neonatal enterostomy nursing after discharge and saves tremendous time and economic cost for the families. Second, WeChat transmits information in various forms such as texts, voice, animations and videos, which is helpful for vivid information communication. It ensures accurate and professional nursing intervention and makes it easier for families to understand and accept knowledge on nursing and feeding. Third, with the WeChat group, the patients’ families can communicate with each other and share their feelings, experience and achievements in nursing and feeding with a team atmosphere of mutual support. In addition, when medical personnel cannot reply in time, families with similar experiences can offer help and share their own experiences. Moreover, with WeChat, the medical personnel can see the photos and videos uploaded by the parents and observe the patients’ stoma conditions and their parents’ nursing status on a daily basis, allowing them to quickly correct nursing errors and offer real-time guidance, which can effectively improve the families’ nursing techniques. In addition, communications via WeChat make nursing work easier, and the the number of patients loss to follow-up decreases.

By implementing continuous nursing based on the WeChat platform for neonates after enterostomy, we achieved more effective nursing care. This study shows that the continuous nursing group dramatically outperformed the traditional nursing group in peristomal skin health. After discharge, the continuous nursing group replaced ostomy bags much less frequently than the traditional nursing group, which not only reduced irritation to the peristomal skin but also reduced household expenses. This indicates that with continuous nursing based on the WeChat platform, medical personnel can help patients’ families tackle nursing problems at home in a timely fashion, the families can better understand the methods of nursing and feeding, and accordingly, the peristomal skin complications decreased.

During the inpatient stay, most mothers are not in contact with their sick baby because it is a Chinese tradition for them to engage in postpartum confinement, but the mothers are generally the main caregivers for home nursing. Therefore, the majority of the patients’ mothers know very little about stoma care. For most families, especially mothers, this will cause a heavy psychological burden, and some may even lose confidence and suffer from depression [23]. Given that the rural medical level in China is low and most rural medical centers are unable to provide professional stoma nursing or address problems in a timely fashion, the families are even more inclined to experience anxiety and negative feelings.

Taking this into consideration, after the patients are discharged, we can guide the parents via WeChat to help them master the necessary nursing knowledge and skills as soon as possible. Meanwhile, WeChat can offer support and visual communication in a timely manner; in cases of emergencies during home nursing care, we can offer guidance immediately. With the WeChat platform, our medical personnel regularly send information on health education and child nutrition to help the families to study, which boosts their confidence in nursing, improves their understanding and skills in enterostomy nursing and elevates their nursing abilities. Thanks to photo and video communications, we are rapidly updated on the patients’ rehabilitation after discharge and can immediately correct mistakes in home nursing. In the meantime, we encourage the families in the WeChat group to communicate and share successful cases and experience, with the purpose of enabling the families to feel the strength of group efforts and enhancing their confidence and hope. By chatting via WeChat, we can also understand the patients’ psychological status in time, listen to them, offer care, guidance and support and ease their negative feelings and anxiety. As revealed by this study, the patients’ depression and anxiety in continuous nursing gourp were noticeably alleviated.

There are very few studies of continuous nursing mode for neonates after enterostomy. Some studies from adults showed the good effects of this new mode of continuous nursing care for adult colostomy patients. The SAS and SDS scores were notably higher in the observation group than in the online training-based continuous caring group [24]. The online social tool WeChat can mediate communication via video, and is real-time, efficient, and nexpensive, and the continuous nursing care mode significantly as shown to decrease colostomy complications significantly [25]. Consistent with the above study, our study showed that continuous nursing based on WeChat could effectively improve the quality of life of neonates after enterostomy.

This study has its limitations. Patients who were incapable of independent reading, had no communication device or were blocked from information communication via WeChat were excluded from this study, which incurs selection bias. Moreover, we were unable to perform subgroup analysis to study different parents based on their scores. In a follow-up study, we will further improve the nursing model, expand our sample size, and conduct a subgroup study to analyse in greater depth the effects of continuous nursing based on the WeChat platform for neonates after enterostomy.

## Conclusion

Continuous nursing based on the WeChat platform extends the high-level and high-quality nursing service and psychological support available in hospitals to patients’ families, which can effectively improve the nursing quality, ease the anxiety and depression of the families, and enhance the quality of life of neonates after enterostomy. The application of the WeChat platform makes continuous nursing more convenient and facilitates face-to-face communication.

## Data Availability

The datasets generated and/or analysed during the current study are not publicly available due to privacy of respondents. Please contact the corresponding author for more information.

## Abbreviations

DET scale: Discoloration, erosion/ulceration and tissue overgrowth scale
SAS: Self-rating anxiety scale
SDS: Self-rating depression scale
